# Myelopathy following intrathecal chemotherapy in a patient with acute lymphoblastic leukemia: a case report

**DOI:** 10.3389/fonc.2026.1851172

**Published:** 2026-07-29

**Authors:** Jiao Li, Xuehan Mao, Fan Yu, Yuehua Huang, Yanying Wang, Jingxian Li, Lihong Li, Bianhong Wang

**Affiliations:** Department of Hematology, Beijing Tsinghua Changgung Hospital, School of Clinical Medicine, Tsinghua Medicine, Tsinghua University, Beijing, China

**Keywords:** acute lymphoblastic leukemia, central nervous system relapse, dorsal column, intrathecal chemotherapy, magnetic resonance imaging, myelopathy, neuro-oncology, neurotoxicity

## Abstract

Intrathecal chemotherapy is a standard treatment for central nervous system (CNS) involvement in acute lymphoblastic leukemia (ALL). However, it may lead to rare but severe neurotoxicity, including chemotherapy-induced myelopathy. We report a 58-year-old female with B-cell ALL who developed progressive myelopathy approximately one month after completing seven cycles of intrathecal chemotherapy for CNS relapse. She presented with bilateral lower limb weakness, sensory disturbance below the T4 level, and urinary retention. Spinal cord MRI showed T2-weighted hyperintense signals in the dorsal columns at C7–T2 without abnormal enhancement. Systemic inflammatory, autoimmune, infectious, metabolic, vascular, and leukemic infiltration etiologies were comprehensively excluded. The diagnosis of intrathecal chemotherapy-induced myelopathy was confirmed. Despite discontinuation of intrathecal chemotherapy and supportive treatment, neurological deficits remained partially irreversible at the 3-month follow-up. This case highlights that intrathecal chemotherapy-related myelopathy is a severe and potentially irreversible complication in patients with ALL undergoing CNS-directed therapy. Characteristic MRI findings and thorough differential diagnosis are essential for early recognition. Close neurological monitoring should be strengthened in neuro-oncological patients receiving intrathecal chemotherapy.

## Introduction

Central nervous system (CNS) involvement is a common and critical issue in the management of acute lymphoblastic leukemia (ALL). Intrathecal chemotherapy has become the cornerstone of prophylaxis and treatment for CNS leukemia, as it can effectively bypass the blood-brain barrier and achieve high local drug concentrations (1–3). Commonly used agents include methotrexate, cytarabine, and dexamethasone. Despite significant survival benefits, neurological complications are increasingly recognized, among which myelopathy represents a rare but potentially devastating neurotoxic adverse event (4–6).

Intrathecal chemotherapy-induced myelopathy (ICIM) is rare, with an estimated incidence of <1-3% in adults receiving repeated intrathecal chemotherapy for ALL. Major risk factors include older age (≥55 years), higher cumulative intrathecal methotrexate/cytarabine doses, frequent administration (twice weekly), and impaired blood-spinal cord barrier integrity (6, 7).

Intrathecal chemotherapy-induced myelopathy (ICIM) often presents subacutely with limb weakness, sensory disturbances, and bowel or bladder dysfunction, typically occurring weeks after drug administration (8–10). The typical radiological feature is T2-weighted hyperintensity in the dorsal columns of the spinal cord (11–13). Diagnosis relies on a clear temporal association with intrathecal therapy and rigorous exclusion of alternative etiologies, including infection, autoimmune myelitis, metabolic disorders, vascular diseases, and direct leukemic infiltration (14, 15). At present, no standardized targeted therapy is available, and the main interventions include immediate cessation of the causative agents and supportive care (12–14).

Here, we report a typical case of ICIM in a patient with ALL after intrathecal chemotherapy for isolated CNS relapse, aiming to improve clinical awareness, early diagnosis, and management of this severe neuro-oncological complication.

## Case presentation

A 58-year-old woman was diagnosed with B-cell ALL in November 2023.She received standard adult B-cell ALL induction chemotherapy (VDP regimen: vincristine, daunorubicin, prednisone) plus high-dose methotrexate (3 g/m²) for CNS prophylaxis, followed by seven cycles of intrathecal chemotherapy (methotrexate 15 mg, cytarabine 40 mg, dexamethasone 5 mg). Following induction, she achieved complete bone marrow remission and received four cycles of consolidation chemotherapy (high-dose cytarabine). Maintenance chemotherapy was initiated in May 2024 and continued until August 2025.

There was no family history of hematological malignancies or inherited neurological disorders in her first-degree relatives. The patient was retired, with no history of tobacco or alcohol abuse or chronic psychiatric illness. Her only comorbidity was mild hypertension well controlled with oral medication. Germline genetic testing at initial ALL diagnosis revealed no pathogenic germline variants. As illustrated in timeline **Table 1**, induction and consolidation chemotherapy yielded complete bone marrow remission, with no severe organ toxicities observed during prior systemic anti-leukemia treatment before isolated CNS relapse.

**Table 1:** Timeline of key clinical events.

Time Point	Clinical Events & Interventions
Nov 2023	Diagnosis of B-cell ALL; induction chemotherapy (VDP regimen) + high-dose methotrexate (3 g/m²) + 7 cycles of intrathecal chemotherapy (MTX 15 mg, Ara-C 40 mg, DEX 5 mg)
May 2024	Complete bone marrow remission achieved; consolidation chemotherapy (4 cycles of high-dose cytarabine); maintenance chemotherapy initiated
Aug 2025	Isolated CNS relapse (neck pain, vomiting, dizziness; CSF flow cytometry: 74% immature lymphocytes; normal bone marrow); 7 cycles of twice-weekly intrathecal chemotherapy
Late Sep 2025	1 month after last intrathecal injection; onset of progressive myelopathy (bilateral lower limb weakness, T4 sensory level, urinary retention)
Sep 2025	Cervical spine MRI: C7–T2 dorsal column T2 hyperintensity, no enhancement
Dec 2025	3-month follow-up; persistent lower limb weakness (MRC grade 3/5), partial improvement of urinary retention

In August 2025, she developed persistent neck pain, non-projectile vomiting, and dizziness. Cerebrospinal fluid (CSF) flow cytometry detected 74% immature lymphocytes, whereas bone marrow evaluation showed no leukemic infiltration, confirming isolated CNS relapse. At the time of isolated CNS relapse, no systemic chemotherapy or radiotherapy was administered; treatment consisted solely of twice-weekly intrathecal chemotherapy (methotrexate 15 mg, cytarabine 40 mg, dexamethasone 5 mg) for seven doses, until CSF minimal residual disease was undetectable.

Approximately one month after the last intrathecal injection, the patient developed progressive bilateral lower limb weakness (Medical Research Council [MRC] grade 3/5; CTCAE grade 3 motor neuropathy), inability to stand or walk independently, and urinary retention. Neurological examination revealed diminished pinprick sensation below the T4 level, reduced lower limb tendon reflexes, and bilateral positive Babinski signs. Cerebellar examination (finger-nose test, heel-shin test, gait assessment) was normal, with no ataxia or dysmetria. Laboratory examinations showed normal complete blood count and serum biochemistry. Autoimmune markers including ANA, ENA, and ANCA were negative. CSF analysis revealed normal cell count, protein, and glucose levels; polymerase chain reaction tests for BK virus and JC virus were negative.

**Figure 1:**
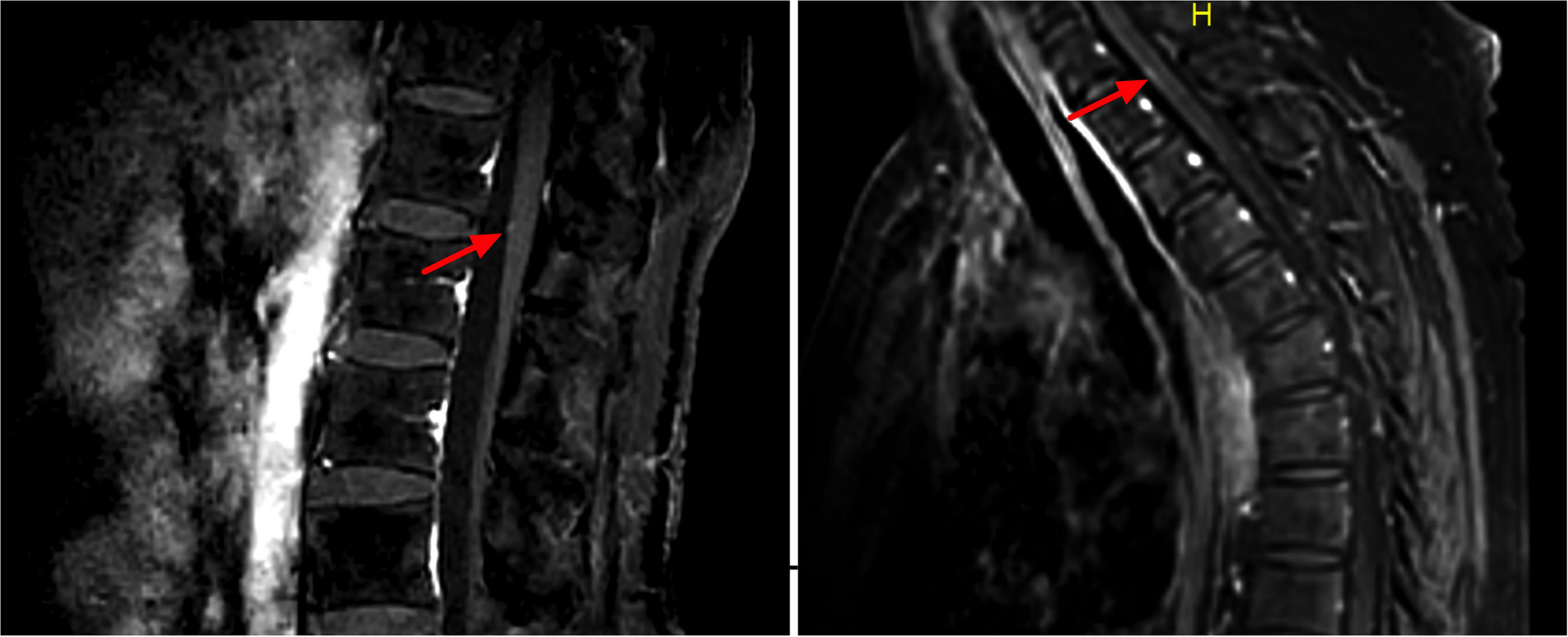
Cervical spine MRI of the patient. Nov 2023: Diagnosis of B‑cell ALL; induction chemotherapy (VDP regimen) + high‑dose methotrexate (3g/m²) + 7 cycles of intrathecal chemotherapy (MTX 15mg, Ara‑C 40mg, DEX 5mg). May 2024: Complete bone marrow remission achieved; consolidation chemotherapy (4 cycles of high‑dose cytarabine); maintenance chemotherapy initiated. Aug 2025: Isolated CNS relapse (neck pain, vomiting, dizziness; CSF flow cytometry: 74% immature lymphocytes; normal bone marrow); 7 cycles of twice‑weekly intrathecal chemotherapy Late Sep 2025: 1 month after last intrathecal injection; onset of progressive myelopathy (bilateral lower limb weakness, T4 sensory level, urinary retention) Sep 2025: Cervical spine MRI: C7-T2 dorsal column T2 hyperintensity, no enhancement. Dec 2025: 3‑month follow‑up; persistent lower limb weakness (MRC grade 3/5), partial improvement of urinary retention

Cervical spine MRI demonstrated symmetric T2-weighted hyperintensity confined to the dorsal columns from C7 to T2, with preserved T1 signal and no post-contrast enhancement. The pattern was consistent with the classic “dorsal column sign” of intrathecal chemotherapy-induced myelopathy (**Figure 1**).

T2-weighted sagittal and axial sequences show characteristic symmetric hyperintensity in the dorsal spinal cord at C7–T2 (red arrows), without contrast enhancement. All alternative causes were systematically excluded, and the diagnosis of intrathecal chemotherapy-induced myelopathy was confirmed (**Table 2**). The patient received vitamin B12 and folic acid supplementation, but neurological symptoms showed no obvious improvement. At the 3-month follow-up, bilateral lower limb strength remained grade 3/5, and urinary retention was slightly improved with partial voiding assistance (**Table 1**).

**Table 2:** Structured differential diagnosis of myelopathy.

Differential diagnosis	Key features	Exclusion basis
Infectious myelitis (viral/BK/JC virus)	Fever, CSF pleocytosis, positive viral PCR	Normal CSF cell count; negative BK/JC PCR
Autoimmune/inflammatory myelopathy	Elevated autoimmune markers, longitudinal myelitis	ANA/ENA/ANCA negative; short-segment dorsal column involvement
Metabolic/nutritional myelopathy	B12/folate deficiency, combined degeneration	Normal serum B12 and folate levels
Vascular myelopathy (spinal infarction)	Acute onset, anterior cord syndrome	Subacute onset; normal vascular imaging
Leukemic spinal cord infiltration	Enhancing lesions, CSF blasts	No enhancement; negative CSF flow cytometry
Intrathecal chemotherapy-induced myelopathy	Subacute onset, dorsal column T2 hyperintensity	Temporal association; classic MRI findings

- Nov 2023: Diagnosis of B-cell ALL; induction chemotherapy (VDP regimen) + high-dose methotrexate (3g/m²) + 7 cycles of intrathecal chemotherapy (MTX 15mg, Ara-C 40mg, DEX 5mg).
- May 2024: Complete bone marrow remission achieved; consolidation chemotherapy (4 cycles of high-dose cytarabine); maintenance chemotherapy initiated.
- Aug 2025: Isolated CNS relapse (neck pain, vomiting, dizziness; CSF flow cytometry: 74% immature lymphocytes; normal bone marrow); 7 cycles of twice-weekly intrathecal chemotherapy.
- Late Sep 2025: 1 month after last intrathecal injection; onset of progressive myelopathy (bilateral lower limb weakness, T4 sensory level, urinary retention).
- Sep 2025: Cervical spine MRI: C7-T2 dorsal column T2 hyperintensity, no enhancement.
- Dec 2025: 3-month follow-up; persistent lower limb weakness (MRC grade 3/5), partial improvement of urinary retention.

### Patient perspective

The patient reported persistent lower limb weakness and urinary difficulty, which significantly impacted daily mobility and independence. She expressed concern about long-term functional recovery despite supportive interventions.

## Discussion

Intrathecal chemotherapy is indispensable for CNS-directed therapy in ALL, but it carries a definite risk of neurotoxicity. ICIM is a rare, severe, and often irreversible complication associated with methotrexate and cytarabine (6, 11). The clinical presentation in this patient was highly typical: subacute progression of lower limb weakness, sensory impairment, and urinary retention occurring approximately one month after intrathecal chemotherapy.

The MRI finding of T2 hyperintensity in the dorsal columns at C7–T2 is a hallmark of ICIM and has been widely reported in adult patients with leukemia (10, 12, 14). This pattern is thought to result from preferential toxic damage to the dorsal columns by chemotherapeutic agents distributed within the subarachnoid space.

### Causative agent analysis

Methotrexate is the most likely causative agent, as it is well-documented to induce selective dorsal column injury via direct oligodendrocyte and axonal toxicity. Cytarabine may potentiate neurotoxicity, while dexamethasone is not associated with this complication. This combination of agents is known to increase the risk of myelopathy in patients receiving repeated intrathecal chemotherapy (7).

The pathogenesis of ICIM is not fully understood but is mainly attributed to direct neurotoxicity of methotrexate and cytarabine, which damage oligodendrocytes and axons, leading to demyelination and axonal degeneration (8, 11). Disruption of the blood-spinal cord barrier and individual genetic susceptibility may also contribute to the development of this complication.

Diagnosis requires rigorous exclusion of other causes commonly seen in neuro-oncological patients, including acute disseminated encephalomyelitis, viral myelitis, metabolic myelopathy, spinal cord infarction, and direct leukemic infiltration (9, 13). Our patient had no evidence of active infection, autoimmune disease, metabolic disturbance, or recurrent leukemia, supporting the diagnosis of ICIM.

Currently, no specific effective treatment is available for ICIM. Prompt discontinuation of intrathecal chemotherapy and supportive care remain the main clinical strategies (11–13). Early recognition and intervention are critical to prevent further neurological deterioration. The prognosis is generally poor, with most patients experiencing persistent neurological deficits, as observed in our case.

### Strengths and limitations

#### Strengths

This case provides a classic example of ICIM after isolated CNS relapse treated with intrathecal triple therapy alone, confirming direct drug toxicity. High-quality labeled MRI and structured differential diagnosis enhance educational value.

#### Limitations

Single case report; lack of genetic testing for predisposition; no long-term (>6 months) follow-up data.

This case underscores the importance of close neurological monitoring in patients with leukemia receiving intrathecal chemotherapy for CNS involvement. Characteristic clinical manifestations and typical MRI features can facilitate early diagnosis. Clinicians in neuro-oncology, hematology, and neurology should maintain high vigilance for this rare but serious complication.

## Conclusion

Intrathecal chemotherapy can induce severe, irreversible myelopathy in patients with ALL and CNS relapse. The condition presents with subacute neurological deficits and characteristic dorsal column T2 hyperintensity on spinal MRI. Thorough exclusion of alternative etiologies is essential for accurate diagnosis. Close neurological monitoring during and after intrathecal therapy is strongly recommended in neuro-oncological practice.

## Data Availability

The datasets presented in this article are not readily available because of ethical and privacy restrictions. Requests to access the datasets should be directed to the corresponding author.
